# Effects of short-term ingestion of Russian tarragon prior to creatine monohydrate supplementation on anaerobic sprint capacity: a preliminary investigation

**DOI:** 10.1186/1550-2783-9-S1-P7

**Published:** 2012-11-19

**Authors:** Mike Greenwood, Jonathan Oliver, AR Jagim, AC Sanchez, K Kelley, Elfego Galvan, James Fluckey, S Riechman, Ralf Jäger, M Purpura, I Pischel, Richard B Kreider

**Affiliations:** 1 Department of Health and Kinesiology, Exercise and Sport Nutrition Laboratory, Texas A&M University, College Station, TX 77843, USA; 2 Increnovo LLC, Milwaukee, WI 53202, USA; 3 PhytoLab GmbH & Co. KG, Dutendorfer Straße 5-7, 91487 Vestenbergsgreuth, Germany

## Background

The improvement in anaerobic exercise capacity associated with supplementation with creatine monohydrate (CrM) has been well established. Extracts of Russian Tarragon (RT) have been reported to produce anti-hyperglycemic effects [1] and influence plasma creatine levels during the ingestion of CrM [2]. Theoretically, RT ingestion may enhance creatine retention and thereby promote greater ergogenic benefit compared to CrM supplementation alone. The purpose of this study was to determine if short-term, low-dose aqueous RT extract ingestion prior to CrM supplementation influences anaerobic sprint performance.

## Methods

In a double-blind, randomized, and crossover manner; 9 untrained males (20±1 yrs; 180±11 cm; 79.9±14 kg) ingested 500 mg of aqueous Tarragon extract (*Finzelberg*, *Andernach*, *Germany*) or 500 mg of a placebo (P) 30-minutes prior to ingesting 5 g of CrM (*Creapure^®^*, *AlzChem AG*, *Germany*) (CrM+RT). Subjects ingested the supplements two times per day (morning and evening) for 5-days and then repeated the experiment after a 6-week wash-out period. Subjects performed two 30-second Wingate Anaerobic Capacity (WAC) tests at baseline, days 3 and 5 of supplementation protocol on an electronically braked cycle ergometer (*Lode*, *Netherlands*) interspersed with 3 minutes rest for determination of peak power (PP), mean power (MP), and total work (TW). Data were analysed by repeated measures MANOVA on 9 subjects who completed both trials. Data are presented as changes from baseline after 3 and 5 days for the CrM+P and CrM+RT groups, respectively.

## Results

Absolute MP (9.2±57, 34.5±57 W; p=0.02), percent change in MP (2.5±11, 6.7±10%; p=0.03), absolute TW (274±1,700, 1,031±1,721 J; p=0.02), and percent change in TW (2.5±11, 6.6±10 %; p=0.03), increased over time in both groups. No significant time effects for both groups were observe in changes from baseline in absolute PP (-15.3±377, -65.7±402 W; p=0.73) or percent change in PP (1.8±21, -1.2±24 %; p=0.82). No significant differences were observed between CrM+P and CrM+RT groups in day 0, 3, or 5 PP (CrM+P 1,472±451, 1,435±182, 1,380±244; CrM+RT 1,559±214, 1,565±398, 1,519±339 W; p=0.92), MP (CrM+P 591±94, 599±89, 643±83; CrM+RT 590±103, 601±78, 608±96 W; p=0.27), or TW (CrM+P 17,742±2,822, 17,970±2,663, 19,264±2,482; CrM+RT 17,706±3,098, 18,029±2,339, 18,246±2,888 J; p=0.28).

## Conclusions

Results suggest as little as 5g CrM taken twice daily for 3-5 days can improve MP and TW by 2-7%. However, results of this preliminary study indicate that ingesting RT 30-min prior to CrM supplementation had no additive effects on anaerobic sprint capacity in comparison to ingesting CrM with a placebo. Additional research is needed to examine whether ingestion of larger amounts of CrM in order to reduce variability, or larger amounts, changes in nutrient timing or increased duration of RT supplementation prior to and/or in conjunction with CrM ingestion would influence the ergogenic benefits of creatine supplementation.
